# Utility of bone SPECT/CT to identify the primary cause of pain in elderly patients with degenerative lumbar spine disease

**DOI:** 10.1186/s13018-019-1236-4

**Published:** 2019-06-20

**Authors:** Satoshi Kato, Satoru Demura, Hidenori Matsubara, Anri Inaki, Kazuya Shinmura, Noriaki Yokogawa, Hideki Murakami, Seigo Kinuya, Hiroyuki Tsuchiya

**Affiliations:** 1 Department of Orthopaedic Surgery, Graduate School of Medical Sciences, Kanazawa University, 13-1 Takara-machi, Kanazawa, 920-8641 Japan; 2 Department of Nuclear Medicine/Biotracer Medicine, Graduate School of Medical Sciences, Kanazawa University, 13-1 Takara-machi, Kanazawa, 920-8641 Japan

**Keywords:** Bone SPECT/CT, Degenerative lumbar spine, Identification, Low back pain, Pain generator

## Abstract

**Background:**

Diagnosis of the cause of low back pain in the presence of degenerative spine disease using conventional imaging techniques, especially in elderly individuals, is challenging. Our aim was to describe our use of bone scintigraphy with single photon emission computed tomography (bone SPECT/CT) in the assessment of low back pain in elderly patients with degenerative lumbar spine disease, underlining the clinical utility of bone SPECT/CT imaging in this clinical population to inform diagnosis and treatment.

**Methods:**

Between January 2016 and December 2017, we used bone SPECT/CT to successfully identify the cause of low back pain in five elderly patients. All patients had been scheduled for extensive spinal fusion surgery based on conventional imaging (plain radiography, computed tomography, and magnetic resonance).

**Results:**

After diagnosis using bone SPECT/CT, three patients underwent spinal fusion at 1–2 levels with specific degenerative disk and joint disease, with the other two patients successfully treated using a conservative approach for a non-traumatic insufficiency fracture of the endplate of the L4 vertebral body and a fracture of the transverse process of L3. Clinically meaningful decrease in pain and fracture healing were obtained with conservative treatment.

**Conclusion:**

Bone SPECT/CT was useful to identify the specific cause of pain in elderly patients with lumbar degenerative disease and to provide appropriate treatment, avoiding the unnecessary use of invasive spinal fusion surgery. Therefore, the clinical utility of bone SPECT/CT is potentially high as it improves diagnosis and lowers the risk of inappropriate invasive spinal surgery.

## Background

Low back pain (LBP) is a major health problem, which is associated with substantial economic and social costs [1]. Among elderly adults, LBP is the most frequently reported musculoskeletal condition and the third most frequently reported symptom of any kind [2, 3]. Establishing a clear diagnosis of LBP is essential to providing appropriate treatment early and improving outcomes [4, 5]. However, the association between abnormal findings on traditional imaging techniques (plain radiography, computed tomography (CT), and magnetic resonance (MR)), clinical symptoms, and exact structure(s) involved is not straightforward, with multiple factors likely to be implicated to different degrees. Moreover, in many elderly individuals, the diagnosis of degenerative spinal disease is a non-specific finding of LBP, as degenerative changes are commonly identified in asymptomatic individuals [6, 7].

The recent introduction of bone scintigraphy with single photon emission computed tomography (bone SPECT/CT) as an imaging modality allows assessment of both morphology and physiology in a single study. CT images provide a precise anatomical localization of the site(s) of radiotracer uptake, despite spinal degenerative changes, which improves the accuracy and specificity of the diagnosis of LBP [8, 9]. CT images also provide additional structural detail about anatomical structures within the imaged volume in which there is no active uptake of radiotracer and, therefore, are likely to not be implicated in the LBP.

Our aim in this study was to describe our effective use of bone SPECT/CT to identify the cause of pain for a series of five patients with LBP, with concomitant degenerative disk and joint disease, which avoided unnecessary extensive spinal fusion surgery.

## Methods

The institutional review board of our university approved this study. Between January 2016 and December 2017, we used bone SPECT/CT to identify the primary cause of pain among five patients with degenerative or aging-associated disorders of the lumbar spine (Table 1). All five patients presented with severe LBP, which had been incorrectly diagnosed based on plain radiographs, MR, and CT imaging obtained at our institution or others. Moreover, all five patients were scheduled for extensive spinal fusion, which proved to be unnecessary. The use of bone SPECT/CT allowed us to confirm the following causes of LBP in these five patients. Three patients had discopathy and facet joint arthropathy, which were confirmed with an analgesic block before surgery. One patient had a non-traumatic spinal fracture of the endplate of the L4 vertebral body and the other had transverse process fracture of L3. The fractures were successfully treated by conservative methods.

**Table 1:** Patient characteristics

Patient no.	Age, sex	Symptoms	Initial diagnosis	Final diagnosis	Primary treatment	NRS before treatment	NRS after treatment
1	68, F	Severe LBP	Adult spinal deformity	Degenerative discopathy	Selected spinal fusion (two levels)	8	0
2	72, F	Severe LBP	Adult spinal deformity	Degenerative discopathy and facet joint arthropathy	Selected spinal fusion (two levels)	8	2
3	77, F	Severe LBP and mild right LE numbness	Adult spinal deformity	Degenerative discopathy (adjacent segment disease after spinal fusion)	Selected spinal fusion (one level)	9	3
4	74, F	Severe LBP and mild left LE pain	Adult spinal deformity	Insufficiency fracture of the end-plate of the L4 vertebral body	Lumbar orthosis	9	4
5	67, F	Severe LBP	Adjacent segment disease after spinal fusion	Insufficiency fracture of the L3 transverse process	Lumbar orthosis	8	2

*F* female, *NRS* 11-point numerical rating scale, *LBP* low back pain, *LE* lower extremity

SPECT/CT was conducted on a hybrid SPECT/CT system with a built-in CT component (Discovery NM/CT 670; GE Healthcare, Waukesha, WI, USA) after injection of a standardized activity of 740 MBq 99mTc-MDP (99m Tc-methylene diphosphonate, TechneTM MDP Injection; FUJIFILM RI Pharma, Tokyo, Japan). After 3 h, whole-body images, SPECT (matrix 512 × 512 mm, axial range 40 cm, collimators adapted to body contour) and CT images (matrix 512 × 512 mm, FOV 50 cm, 120 kV, 100–440 mAs [automated dose modulation], rotation time 0.5 s, slice thickness 2.5 mm) were acquired. CT images were reconstructed iteratively using AsIRTM (Adaptive Statistical Iterative Reconstruction, GE Healthcare) with a slice thickness of 2.5 mm in all three planes. SPECT and CT images were fused by an automated software algorithm on a dedicated diagnostic workstation (Advantage Workstation 4.5, GE Healthcare).

For analysis, the intensity of the LBP was evaluated using an 11-point numerical rating scale (NRS), with “0” indicating no pain and “10” the worst pain. The NRS score was obtained before treatment and at the last follow-up visit, at a minimum of 1 year. Data are presented as means and standard deviations. Pre- to post-treatment change in the NRS score was evaluated using paired *t* tests, with the level of significance level set at 0.05. Analyses were performed using SPSS software (version 19.0 for Windows; SPSS Inc., Chicago, Illinois).

## Results

The NRS scores and the final diagnosis based on the bone SPECT/CT are reported in Table 1. Overall, including the two patients treated conservatively, we achieved a decrease in the NRS pain score from 8.4 ± 0.5, before treatment, to 2.2 ± 1.3, post-treatment (*P* < 0.05). In contrast to the multilevel spinal fusion that had been planned for all five patients, based on the specific diagnosis and localization of the cause of pain provided by SPECT/CT, all patients were successfully treated with minor spinal surgery (segmental spinal fusion) or conservative treatment. For patients treated using a conservative approach, one had a diagnosis of non-traumatic insufficiency fracture of the endplate of the L4 vertebral body and the other of transverse process fracture of L3. Significant pain relief was obtained in these two patients, with fracture healing. The clinical presentation, assessment, and treatment for three representative cases are provided below as examples of the clinical utility of bone SPECT/CT.

### Case 1 (patient 1 in Table 1 and Fig. 1)

**Fig. 1:**
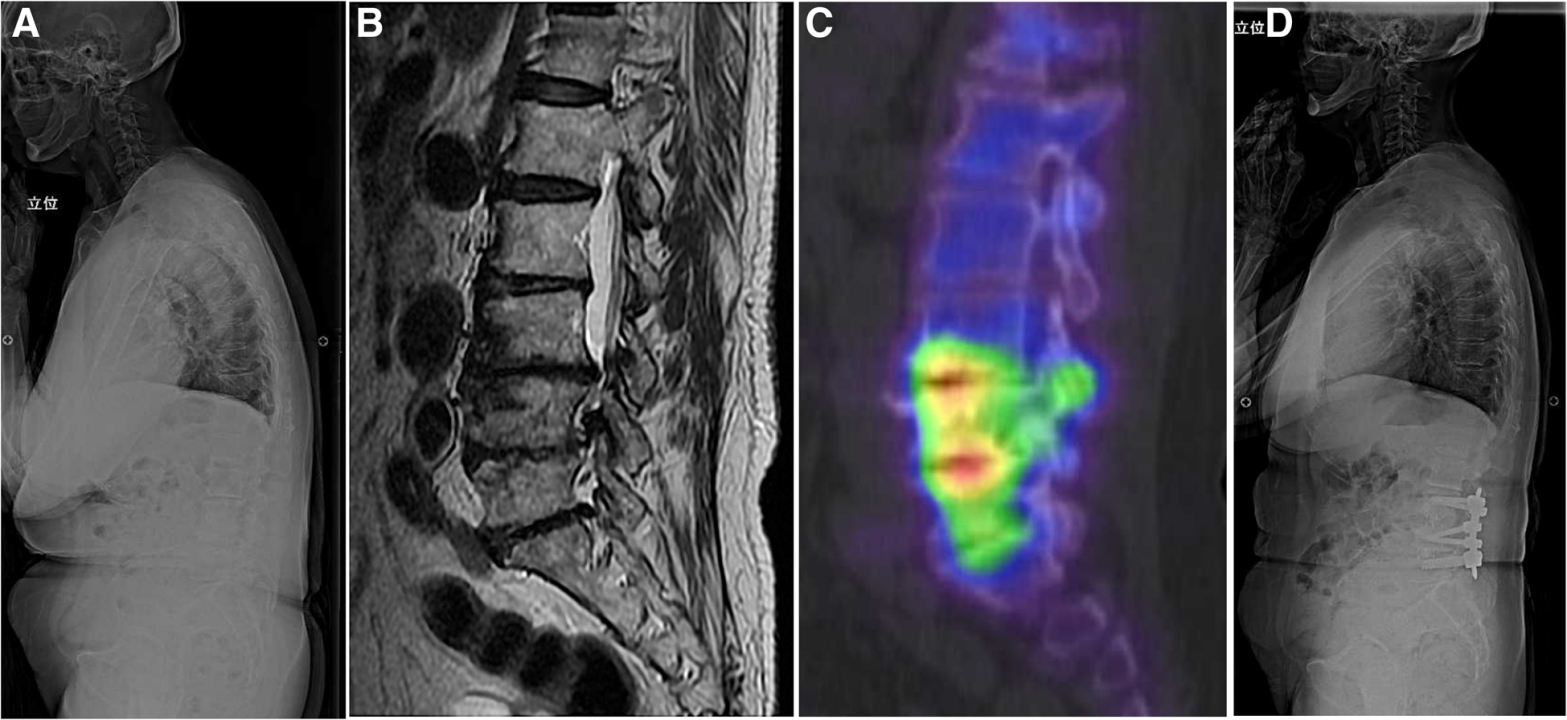
A 68-year-old woman with degenerative lumbar discopathy. **a** Lateral view of radiograph showing adult spinal deformity with a sagittal imbalance. **b** T2-weighted MR sagittal image of the lumbar spine without remarkable findings indicating the site of pain-causing pathology. **c** Bone SPECT/CT image of the lumbar spine showing a hotspot in the vertebral disks of L3/4 and L4/5. **d** Lateral view radiograph after oblique lumbar interbody fusion and percutaneous pedicle screw fixation. MR, magnetic resonance; SPECT/CT, bone scintigraphy with single photon emission computed tomography

The patient was a 68-year-old woman with severe LBP, which had deteriorated over the past 5 years. She was unable to walk without support for < 100 m and experienced restrictions in her activities of daily living. Based on clinical symptoms and radiographic findings (Fig. 1a), adult spinal deformity (ASD) with sagittal imbalance was initially suspected, and corrective spinal fusion surgery, extending from the thoracic spine to the pelvis, was planned. No remarkable findings were observed in MR images (Fig. 1b). Bone SPECT/CT of the lumbar spine revealed a localized radiotracer uptake in the endplates of the L3/4 and L4/5 intervertebral disk (Fig. 1c). A discoblock of L3/4 and L4/5 dramatically relieved her LBP for several days. Based on this finding, the patient underwent an oblique lumbar interbody fusion of L3/4 and L4/5, with percutaneous pedicle screw fixation, without direct correction and decompression (Fig. 1d). The severity of her LBP was sufficiently relieved after surgery which improved her activities of daily living, which had been maintained for 3 years after surgery.

### Case 2 (patient 4 in Table 1 and Fig. 2)

**Fig. 2:**
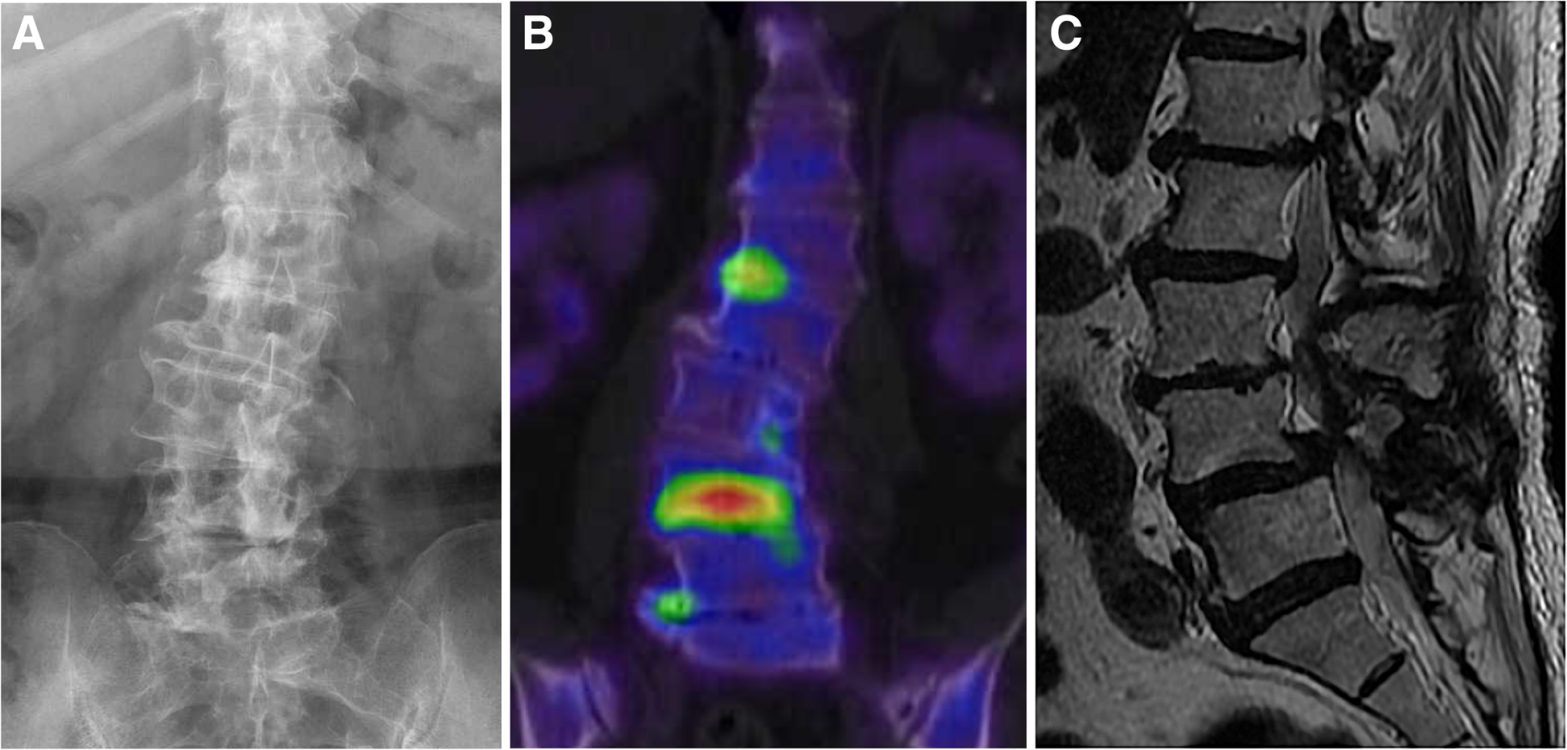
A 74-year-old woman with an insufficiency endplate fracture of L4 vertebral body. **a** Anterolateral view radiograph showing a degenerative lumbar scoliosis. **b** Bone SPECT-CT image of the lumbar spine showing a hotspot at the lower endplate of the L4 vertebral body. **c** T2-weighted MR sagittal image of the lumbar spine without remarkable findings indicating the site of pain-causing pathology. MR, magnetic resonance; SPECT/CT, bone scintigraphy with single photon emission computed tomography

The patient was a 74-year-old woman with a degenerative kyphoscoliosis of the lumbar spine (Fig. 2a), who had been experiencing moderate LBP for several years. However, her LBP had gradually worsened over the past 2 months, without any trauma, to the point of limiting her activities of daily living. Based on findings on plain radiographs and CT and MR images, corrective spinal fusion surgery, from the thoracic spine to the pelvis, was planned. However, bone SPECT/CT of the lumbar spine revealed a localized radiotracer uptake in the lower endplate of L4 (Fig. 2b), indicative of a minor fracture. A retrospective review of MR images confirmed the presence of a low-intensity area in the lower endplate of L4 on T1-weighted images and a local high-intensity signal on T2-weighted images (Fig. 2c). These signal changes were difficult to differentiate from the degenerative changes and deformity of the lumbar spine. A lumbar orthosis was applied, with a decrease in LBP and healing of the endplate fracture, although the degenerative lumbar deformity remained, with persisting moderate LBP.

### Case 3 (patient 5 in Table 1 and Fig. 3)

**Fig. 3:**
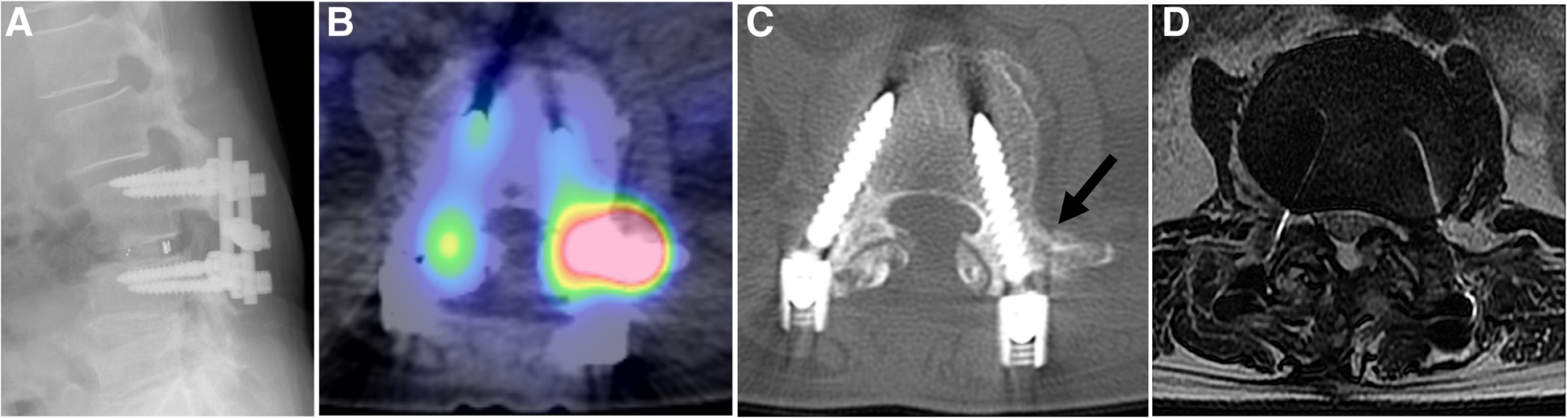
A 67-year-old woman with an insufficiency fracture of the L3 transverse process. **a** Lateral view of radiograph showing an L2 spondylolisthesis after posterior lumbar interbody fusion of L3/4, with adjacent spinal degenerative disease considered the cause of pain based on pain location and imaging assessment using plain radiographs, computed tomography, and magnetic resonance imaging. **b** Bone SPECT/CT image showing a hotspot in the right transverse process of L3, and not in the facet joint of L2/3. **c** On retrospective review of the computed tomography image, the non-traumatic insufficiency fracture of the right transverse process of L3 was identified (black arrow), and the patient was treated using oral steroids for rheumatoid arthritis. **d** T2-weighted MR axial image without findings indicating the insufficiency fracture of the right transverse process of L3. MR, magnetic resonance; SPECT/CT, bone scintigraphy with single photon emission computed tomography

The patient was a 67-year-old woman who underwent a posterior lumbar interbody fusion of L3/4 at another hospital and who was treated using oral steroids for rheumatoid arthritis. She experienced a severe LBP that gradually worsened over the past 1 month, without any trauma. Based on findings on plain radiographs and CT and MR images, L2 spondylolisthesis with sagittal imbalance was identified (Fig. 3a), and extended spinal fusion surgery was considered. However, bone SPECT/CT of the lumbar spine showed a localized radiotracer uptake in the right transverse process of L3 (Fig. 3b). A retrospective review of CT images identified the non-traumatic insufficiency fracture of the right transverse process of L3 (Fig. 3c). The fracture was not identified with MR images (Fig. 3d). A successful decrease in LBP and healing of the endplate fracture was achieved with a conservative treatment using a lumbar orthosis.

## Discussion

We described five cases in which the cause of LBP was accurately identified using bone SPECT/CT, which allowed us to provide specific treatment, successfully improve patients’ symptoms and activities of daily living, and avoid unnecessary extensive multilevel spinal fusion. All five patients were successfully treated with a specific segmental spinal surgical intervention or conservatively.

In the USA, annual spine care-related expenditures are estimated to be over $80 billion [10], with spinal surgeries representing an increasing fraction of this substantial expenditure. Surgical treatment of spinal degenerative disease in adults is an area of specific concern for health care costs, with the number of surgeries related to ASD having more than doubled from 2000 to 2010 [11]. The complex surgical procedures needed to manage ASD are associated with significant resource utilization and high cost; therefore, reducing the number of multilevel spinal fusion procedures [10], as well as lowering the burden of major surgery for patients, would be both cost-effective. Accurate assessment of the cause of pain in patients with degenerative spine disease is a key component to achieving this goal.

Diagnosis of intervertebral discopathy or joint arthropathy continues to pose a challenge to clinicians. Conventional radiological techniques, such as MR and plain CT imaging, dynamic bending films, and planar radionuclide bone scanning, do not consistently provide accurate localization of the source of spinal pain, which is required for diagnosis and targeted treatment. The use of controlled comparative local anesthetic blocks has become an acceptable alternative for confirming a diagnosis in patients with LBP. Recently, the use of hybrid bone SPECT/CT imaging has been introduced into practice to provide accurate localization of the site of radiotracer uptake, which has been successful in improving the accuracy and specificity of malignancy, trauma, infection, and postoperative pseudoarthrosis [8, 12–14]. Based on this success, there has been increasing interest in the possible application of bone SPECT/CT in the evaluation of non-specific musculoskeletal pain [15–17], particularly as SPECT has been shown to provide a more reliable detection and more accurate localization of lesions compared to planar scintigraphy [18]. Compared to MR imaging, bone SPECT/CT has also been shown to provide more important findings to rule out particular causes of LBP [19]. The better accuracy and reliability of bone SPECT/CT in locating the site of LBP compared to other imaging techniques is a particular advantage when we consider the complicated anatomy of the spine, including endplates and transverse processes that are adjacent to vertebral bodies and nearby small joints, as well as the frequent co-existence of multiple spinal pathologies.

In our case series, we describe the clinical utility of combining SPECT with high-resolution structural CT imaging to accurately localize the site of pain-causing pathology, despite the co-existence of spinal degenerative disease, which allowed us to provide targeted and appropriate treatment.

Although MR is considered the gold standard for spinal imaging, marked variability in the interpretation of findings has been reported, with a high prevalence of interpretive differences in radiologists’ report of lumbar spine disease from MR scans performed on the same patient [20]. One of the advantages of bone SPECT/CT is that the radiotracer uptake by an active lesion produces a bright signal, which reduces the risk of missing even a small active lesion, and, when combined to CT, can be accurately localized (Figs. 1, 2, and 3).

A major outcome of our study was the avoidance of extensive multilevel spinal fusion for an inaccurate diagnosis of ASD in all patients. Based on bone SPECT/CT findings, we were able to provide less invasive surgical or conservative treatments, including selective short segment spinal fusion in three patients with degenerative discopathy and/or facet joint arthropathy, and conservative treatment in two patients with insufficiency vertebral fractures. The avoidance of major surgery certainly lowers the risk to patients, including unnecessary operative stress, as well as the cost of LBP treatment. Therefore, considering that the number of surgeries for ASD has dramatically increased, especially in developed countries, bone SPECT/CT may help prevent inadequate surgery for ASD.

This study has some limitations. The sample size was small, and there were no inclusion/extrusion criteria for the selection of participants undergoing bone SPECT/CT to identify the primary cause of pain. The use of bone SPECT/CT should be restricted to patients with severe pain and scheduled for surgery. In addition, it should be applied in cases in which the site of pain-causing pathology was not determined through traditional imaging techniques. The estimated effective radiation dose to a patient undergoing a 99m Tc-bone scan is 2.5 mSv [21]. This is safe and comparable to the annual background radiation dose of 1–2 mSv [22]. Risk and benefit assessment of nuclear imaging was quite necessary for bone SPECT/CT imaging in the evaluation of degenerative musculoskeletal diseases.

## Conclusion

Using bone SPECT-CT, we were able to successfully identify causes of LBP among the elderly patients with degenerative lumbar spine disease, with causes having been overlooked using conventional imaging. Therefore, the clinical utility of bone SPECT/CT is potentially high, improving diagnosis and lowering the risk of inappropriate invasive spinal surgery.

## Data Availability

The datasets during and/or analyzed during the current study are available from the corresponding author on reasonable request.

## Abbreviations

ASD: Adult spinal deformity
Bone SPECT/CT: Bone scintigraphy with single photon emission computed tomography
CT: Computed tomography
LBP: Low back pain
MR: Magnetic resonance
NRS: Numerical rating scale
